# Characteristics of cancer-related fatigue and its correlation with anxiety, depression, and stress-related hormones among Chinese cancer survivors: a cross-sectional study

**DOI:** 10.3389/fonc.2023.1194673

**Published:** 2023-10-26

**Authors:** Shanshan Gu, Yun Xu, Xiaoshu Zhu, Anderson Lam, Danhui Yi, Lutian Gong, Jinghui Wang, Xinyu Guo, Li Fu, Jiyan Shi, Feiye Wang, Ketan Liu

**Affiliations:** ^1^ Oncology Department, Xiyuan Hospital of China Academy of Chinese Medical Sciences, Beijing, China; ^2^ National Institute of Complementary Medicine (NICM) Health Research Institute, Western Sydney University, Penrith, NSW, Australia; ^3^ School of Statistics, Renmin University of China, Beijing, China; ^4^ School of Beijing University of Chinese Medicine, Beijing, China

**Keywords:** cancer-related fatigue, cancer survivors, influencing factors, anxiety, depression, stress-related hormones

## Abstract

**Background:**

Fatigue is a common source of distress for cancer survivors. The severity of cancer-related fatigue varies significantly, which may be due to individual differences in host factors.

**Aim:**

This cross-sectional study aims to explore how demographic, oncological, sociological, psychological, and stress-related hormones levels interact to influence the distinct experiences of fatigue (Cancer-related fatigue [CRF] occurrence and fatigue degree).

**Methods:**

A cross-sectional study carried out at the oncology outpatient and ward department of Xiyuan Hospital of China Academy of Chinese Medical Sciences recruited 306 cancer patients between January 2021 to December 2021. General information, fatigue, psychological factors was evaluated by general information questionnaire, the Revised Piper’s Fatigue Scale-Chinese Version (RPFS-CV), and the self-report Hospital Anxiety and Depression Scale (HADS). Stress-related hormones were measured with chemiluminescent enzyme immunoassay (Zhengzhou Antobio).

**Results:**

306 patients were included, 229 (74.8%) were diagnosed with CRF, including 94 (41.0%) with mild fatigue, 121 (52.8%) with moderate fatigue, and 14 (6.1%) with severe fatigue. Multivariate regression analysis showed that higher depression scores, aldosterone levels may increase the risk of CRF. Patients who are obese (Body mass index ≥ 28 kg/m^2^) may help to reduce the risk of CRF. Other contributing factors for increased levels of fatigue (*p*< 0.05) include being female, having anxiety, depression and high aldosterone levels.

**Conclusion:**

The research suggested that CRF was a common symptom in cancer survivors and pay attention to these influencing factors may help to better identify patients susceptible to fatigue and provide long-term, targeted interventions.

## 1 Introduction

The number of cancer survivors is predicted to reach 20.6 million in 2040 due to advancements in early detection, diagnosis, treatment, and rehabilitation (1). Cancer survivors report that fatigue is a disruptive symptom month or even years after treatment is completed. More than 30% of tumor-free cancer survivors in China report persistent fatigue, even years after finishing treatment (2–4). The NCCN defines cancer-related fatigue (CRF) as a distressing, persistent, subjective sense of physical, emotional, and/or cognitive tiredness or exhaustion related to cancer or cancer treatment that is not proportional to recent activity and interferes with usual functioning (5). The distressing, persistent, multi-dimensional nature of fatigue leads to treatment interruption, decreased quality of life, which makes it difficult for patients to resume “regular” family, work, and life (6–9).

Numerous variables that influence CRF can be broadly categorized into three categories: oncological variables, demographic variables, and psychosocial variables (10, 11). Tumors and anti-tumor therapy are the direct causes of CRF, nevertheless, susceptibilities to exhaustion and levels of fatigue might vary among individuals with the same types of cancer or receiving the same treatments. For instance, one study found that among 67 breast cancer patients receiving simultaneous chemotherapy, 46.3% displayed higher levels of exhaustion and 56.7% displayed lower levels of fatigue (12), while there were similar differences among tumor patients who received other treatments or did not receive treatment. Female, insomniac, depressed, neurotic, and other factors are widely known as possible influencing factors of CRF. In recent years, some scholars have proposed that the difference experience of fatigue may be related to some congenital factors (e.g., SNP genes, cellular aging) or specific social and psychological factors (e.g., childhood abuse, depression history, trait anxiety, catastrophizing, etc.) (3, 13), broadening the researchers’ horizons.

The specific pathological mechanism of CRF has still not been elucidated (14–18), several studies have shown that some factors, such as anxiety, depression, physical and mental stress, may act as persistent stressors to affect the HPA axis and autonomic nervous system, causing neuroendocrine rhythm dysregulation(e.g., diurnal cortisol, aldosterone, adrenocortical hormone, etc.) (19–21). Cortisol has long been considered as a potential predictor of CRF, many clinical studies have demonstrated that CRF is associated with a flat cortisol secretion rhythm (22, 23). In fact, there is evidence that high salivary aldosterone concentrations as a marker of depression and prolonged chronicity, which also has been implicated in CRF (24, 25). Therefore, we conducted a 1-year cross-sectional study to further explore CRF specific or persistent influencing factors and potential predictors, so as to help clinicians identify susceptible populations for CRF and give patients long-term, more targeted intervention strategies.

## 2 Methods

### 2.1 Study design and setting

A cross-sectional survey was carried out in the outpatient department and ward of the Oncology Department of Xiyuan Hospital, China Academy of Chinese Medical Sciences from January 2021 to December 2021. Recruitment of study participants occurred between April 26, 2021, and December 31, 2021. Study protocol registered with the China Clinical Trials Registry (Registration number: ChiCTR2100045404; Registration date: April 14, 2021). Ethical approval was obtained from the Medical Ethics Committee of Xiyuan Hospital, China Academy of Chinese Medical Sciences (Approval number:2021XLA027; Approval date: March 26, 2021), and all subjects signed an informed consent form before being investigated.

### 2.2 Study population

Included patients were aged 18 and above, with clear diagnosis of tumour by biopsy, pathology, or cytology and KPS ≥ 60 points. Exclusion criteria included major traumatic damage such as surgical treatment in the last month, severe combined heart, liver, kidney and other systemic diseases, poor compliance, severe cognitive impairment or psychiatric disorders, inability to complete the scale, and those who are unaware of their disease.

### 2.2 Data measurement

#### 2.2.1 General information

A questionnaire was created to gather general information of patients (including demographic characteristics, oncological characteristics, and sociological characteristics). Demographic characteristics include gender, age, exercise (intensity, frequency, and time), dietary habits (dietary structure, dietary taste), and BMI. Oncological characteristics include tumor type, pathological type, whether tumor-free, metastatic site, tumor stage, stage of disease treatment, whether surgery was performed, previous treatment, current treatment, comorbidities, and KPS. Sociological characteristics include marital status, educational level, fatigue cognition, work nature, work status, and family income.

#### 2.2.2 Fatigue screening, diagnosis, evaluation

##### 2.2.2.1 Filter criteria

Patients who met the inclusion and exclusion criteria were initially assessed using the Visual Analogue Fatigue Scale (VAFS) (26); a score of 0 was defined as non-CRF, and a score of 1 or above was then further diagnosed in accordance with the CRF diagnostic criteria in the ICD-10.

##### 2.2.2.2 Diagnostic criteria

Patients with a VAFS scale screening score of 1 or higher were referred to the 10th International Conference on the Revision of the International Classification of Diseases diagnostic criteria for CRF (ICD-10) (27).

##### 2.2.2.3 Evaluation criteria

The Revised Piper’s Fatigue Scale-Chinese Version (RPFS-CV) scale was used to measure fatigue in patients who satisfied the diagnostic standards for CRF. Patients were classified into three categories of fatigue: mild fatigue (1-3 points), moderate fatigue (4-6 points), and severe fatigue (7–10 points) (28).

#### 2.2.3 Assessment tools

##### 2.2.3.1 The visual analogue fatigue scale

VAFS (26) is mainly used to screen whether the patient is tired. To start, mark a horizontal line on the paper that is 10 cm long with a 0 on the left side and a 10 on the right. A score of 0 indicates no fatigue, a score of 10 complete exhaustion, a score of 1-3 indicates mild fatigue, a score of 4-6 indicates moderate fatigue, and a score of 4-6 indicates severe fatigue. The scale is simple and easy to fill out, and can be used for measurement multiple times. Fatigue is assessed and recorded at 7 a.m., 12 a.m., 1 p.m., and 7 p.m. every day to understand the dynamic changes of CRF and influencing factors of patients in the wakeful state, to help patients manage time reasonably, allocate energy, and improve self-management ability. VAFS score is especially suitable for patients with cancer fatigue and pain.

##### 2.2.3.2 The revised Piper’s fatigue scale-Chinese version

RPFS-CV is a Chinese translation of the original scale that has been validated in China and is extensively utilized in local clinical research (28). It comprises of 24 questions assessing total CRF. Item 1 asks the patient if fatigue is present and, if so, continues with the following questions, item 2 records the duration of the patient ‘s fatigue. Items 3-24 respectively evaluated the four dimensions of fatigue, namely, behavioural/severity (items 3-8), emotion (items 9-13), feeling (items 14-18) and cognition/emotion (items 19-24). Patients can be categorized into four categories of weariness according to the Likert 11 scale: none (0 points), mild (1-3 points), moderate (4-6 points), and severe (7-10 points).

##### 2.2.3.3 Anxiety/depression assessment

The self-report Hospital Anxiety and Depression Scale (HADS) (29) was used to assess the level of anxiety (HADS-A) and depression (HADS-D) during the previous week. The scale is composed of 7 questions each for anxiety (HADS-A) and depression (HADS-D) questions, symptoms were reported on a scale from 0 (not at all) to 3 (most of the time). Scores of 0 to 7 were considered asymptomatic, 8 to 10 indicated a suspicious presence, and 11 to 21 indicated a confirmed presence. In our study, all scores of 8 and above were considered as the presence of anxiety/depressive states.

##### 2.2.3.4 Karnofsky performance Status, body mass index

KPS Index is an assessment tool for functional impairment and prognosis in cancer survivors (30). Ranging from 0 (dead) to 100 (normal activity, healthy), with a high score considered to be between 80 and 100. Based on Chinese Adult Body Weight Standard (31), we divided patients into four groups according to their body weight: lean (BMI< 18.5 kg/m^2^), normal weight (BMI 18.5-23.9 kg/m^2^), overweight (BMI 24.0-27.9 kg/m^2^) and obesity (BMI ≥ 28.0 kg/m^2^).

##### 2.2.3.5 Stress-related hormones

Test items include adrenocortical hormone (ACTH), aldosterone (ALD), renin (Renin), and cortisol (COR). The detection time is the early morning of the day or early morning the following day. A tube of venous blood is collected from the patient in a supine position on an empty stomach. More than 3ml of EDTA-K2 (dipotassium ethylenediaminetetraacetate) is used for anticoagulation (long-headed purple tube). Within half an hour after the sample is collected, the sample is sent to the laboratory. Plasma was separated by centrifugation at 3000g for 10 min at 4°C, and then aliquoted into siliconized polypropylene tubes and stored at −80°C until batched assay. The detection kit was Zhengzhou Autobio Diagnostics Quantitative Assay Kit (chemiluminescent enzyme immunoassay).

#### 2.2.4 Information collection and quality control

Patients who met the inclusion and exclusion criteria were screened by two trained investigators (Shanshan GU and Jinghui Wang). After the investigators obtained informed consent from the patients and signed the informed consent form, patients completed the scales independently or with the assistance of the investigators under the guidance of the investigators and an attending oncologist to ensure the quality of the study.

#### 2.2.5 Sample size calculations


$$n=\frac{Z_{\alpha /2}^{2}p\left(1-p\right)}{\delta^{2}}$$

The purpose of this cross-sectional study was to explore the prevalence of CRF, requiring a two-sided test, α taking 0.05, then Z is 1.96, *p* is the incidence of CRF, the literature reports the incidence of CRF is 60%-100% (5), this study takes 60% incidence, δ for the allowable error, δ take 0.1*p*. Using a 0.05 significance level, 80% power, the sample size required for this investigation is about 256, considering 10-15% incomplete data or other reasons for exclusion, a total of at least 300 cases are proposed to be included. The calculated sample size is sufficient to build a multi-factor logistic regression model.

#### 2.2.6 Data analysis

Data were entered into Epidata software using double entry, and IBM SPSS Statistics 26 software was used for data statistics and analysis. Shapiro-Wilk (S-W) normality test revealed that the distributions of anxiety, depression, and stress-related endocrine hormones differ from a normal distribution. The mean value and standard deviation were calculated for continuous variables and number (*n*) and proportion (%) of participants were reported for categorical measures. The χ2 test was used to compare the differences between the non-CRF and CRF groups on demographic, oncological, and sociological characteristics, the Mann-Whitney U rank sum test was used to compare the differences between groups on anxiety, depression, and serum hormone levels. The Kruskal-Wallis H rank sum test was used to compare the differences between the three groups of mild, moderate, and severe fatigue on demographic, oncological, and sociological characteristics, anxiety and depression, and serum hormone levels. Binary logistic regression and orthogonal logistic regression were established to investigate the specific degree of influence of the factors significant in the analysis of variance on whether suffering from CRF and the degree of fatigue of patients, respectively. Analysis was adjusted by controlling for age, gender, BMI, stage of disease treatment, and previous and current treatment modalities. A two-tailed test with a test level of 0.05 and 95% confidence interval was used, and missing values were filled in using the mean.

## 3 Results

**Figure 1:**
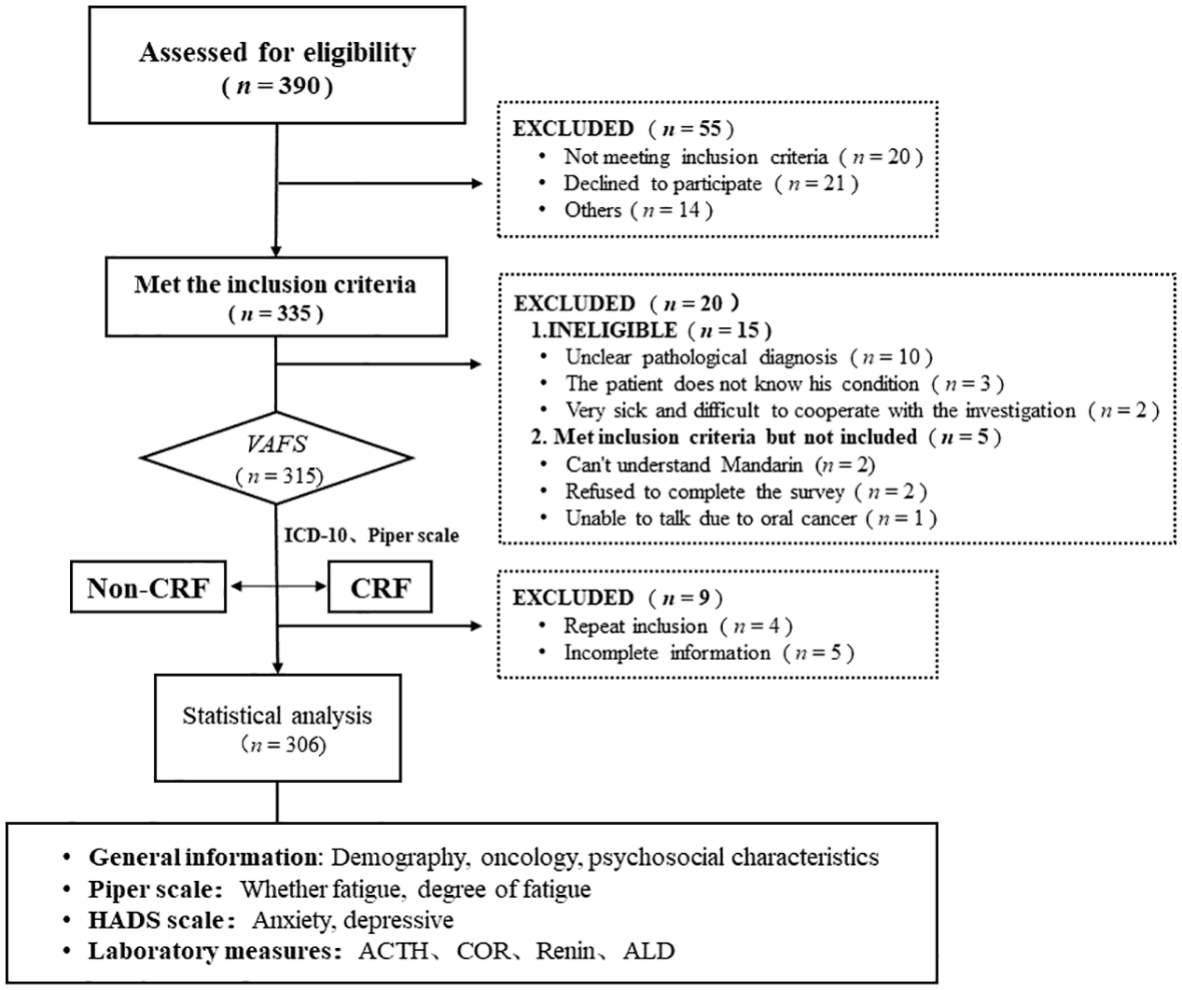
STORBE Diagram.

A total of 390 tumor patients were screened in this study, with 335 tumor patients who met the inclusion criteria, 315 who patients entered fatigue screening or evaluation, and 306 patients who were included in the final statistical analysis (**Figure 1**).

### 3.1 General information

Of the 306 patients, 229 (75.0%) were experiencing CRF. Among CRF patients, those with mild, moderate, and severe fatigue comprised 41.0%, 52.8%, 6.1%, respectively. The median age was 63 (22-89) years, which consisted of 169 males (55.2%) and 137 females (44.8%). Most of the patients (90%) had exercise habits, whereas 41.2% had dietary bias. There were 93 cases (30.4%) of gastrointestinal cancer, 81 cases (26.5%) of lung cancer, 50 cases (16.3%) of breast cancer, 191 cases (62.4%) of patients with tumor survival, 230 cases (75.2%) of III-IV patients, about 60% of patients received anti-tumor therapy, and 111 patients (36.2%) patients were receiving chemotherapy. Thirty-one (10.1%) had no spouse, 32 (10.5%) had heard of CRF, and 14 (4.6%) had actively intervened for fatigue. The total mean score of anxiety and depression were 3.11 ± 3.69, 5.65 ± 4.83 points, respectively. The average level of aldosterone was 162.14 ± 61.56 pg/ml (**Table 1**).

**Table 1:** General characteristics of 306 cancer patients.

(1) Demographic characteristics	Mean ± SD/n (%)
Sex	
Male	169 (55.2)
Female	137 (44.8)
Age (years)	
Median (range)	63 (22-89)
< 30	4 (1.3)
30-50	52 (17.0)
51-70	181 (59.2)
71-90	69 (22.5)
Exercise habits	
No	31 (10.1)
Yes	275 (89.9)
▪ Intensity	
Light	223 (81.1)
Moderate	43 (15.6)
Heavy	9 (3.3)
▪ Frequency	
< 3 times/week	17 (6.2)
3 times/week	194 (70.5)
Irregular	64 (23.3)
▪ Time (min/time)	
< 30	39 (14.2)
30-60	164 (59.6)
> 60	72 (26.2)
Dietary structure	
Balanced diet	180 (58.8)
Dietary bias	126 (41.2)
**(2) *Oncology characteristics***	Mean ± SD*/n* (%)
Cancer type	
Gastrointestinal cancer	93 (30.4)
Lung cancer	81 (26.5)
Breast cancer	50 (16.3)
Other	82 (26.8)
Pathological type	
Adenocarcinoma	146 (47.7)
Squamous cell carcinoma	64 (20.9)
Invasive ductal carcinoma	48 (15.7)
Other/unknown pathology	48 (15.7)
Tumor status	
Tumor-free survival	115 (37.6)
Survival with tumor	191 (62.4)
Metastasis	
Local recurrence	17 (11.8)
Single-site metastasis	63 (43.8)
Two or more sites metastasis	64 (44.4)
Current cancer stage	
I	138 (45.1)
II	159 (52.0)
III	71 (23.2)
IV	159 (52.0)
Unknown	9 (2.9)
Stage of treatment	
Just diagnosed	14 (4.6)
Peri-western medical treatment period	246 (80.4)
Follow-up period	46 (15.0)
Surgery	
Yes	214 (69.9)
No	92 (30.1)
Received anti-tumor therapy	
Yes	182 (59.5)
No	124 (40.5)
Current therapy	
No	14 (4.6)
Chemotherapy	111 (36.2)
Radiotherapy	44 (14.3)
Targeted therapy	25 (8.1)
TCM treatment	112 (36.6)
KPS	
≥80	251 (82.0)
<80	55 (18.0)
BMI	
Lean	20 (6.5)
Normal	160 (52.3)
Overweight	109 (35.6)
Corpulent	17 (5.6)
**(3) *Sociological characteristics***	Mean ± SD*/n* (%)
Marital status	
No spouse	31 (10.1)
Have a spouse	275 (89.9)
Education level	
Elementary	113 (36.9)
Secondary	102 (33.3)
University	91 (29.7)
Fatigue cognition	
▪ Heard of CRF	
Yes	32 (10.5)
No	274 (89.5)
▪ Have actively intervened in CRF	
Yes	14 (4.6)
No	292 (95.4)
Work nature	
Mental work	114 (37.3)
Physical work	95 (31.0)
Other	97 (31.7)
Working status	
Retire	212 (69.3)
Incumbency	61 (19.9)
Other	33 (10.8)
Monthly household income (RMB)	
<5000	71 (23.2)
5000-10000	103 (33.7)
10000-20000	50 (16.3)
>20000	82 (26.8)
(4) Psychological factors	
HADS-A	3.11 ± 3.69
HADS-D	5.65 ± 4.83
(5) Serum hormone level (pg/ml)	
ACTH	35.47 ± 20.18
Renin	13.64 ± 10.33
COR	15.63 ± 19.66
ALD	162.14 ± 61.56
(**6**) ***Fatigue degree ^a^***	3.95 ± 1.63
**Non-CRF**	77 (25.0%)
**CRF**	229 (75.0%)
▪ Mild	94 (30.7%)
▪ Moderate	121 (39.5%)
▪ Severe	14 (4.6%)

SD standard deviation; TCM, Traditional Chinese Medicine; Dietary bias, vegans, high-salt food and meat-based food, etc.; HADS-A, HADS anxiety subscale, HADS-D, HADS depression subscale.

a Scoring based on RPFS-CV scale, none (0 points), mild (1-3 points), moderate (4-6 points), and severe (7-10 points).

### 3.2 Comparison of differences between CRF and non-CRF groups

As shown in **Table 2**, in the general information, dietary structure (*p* = 0.039), KPS (*p*< 0.001), and history of anti-tumor treatment (*p* = 0.036) were significantly associated with the occurrence of CRF. There was no significant difference in the sociological data between non-fatigue and fatigue groups (*p* > 0.05).

**Table 2:** Comparison of differences between non-CRF and CRF in general information.

	Non-CRF (*n* = 77)	CRF (*n* = 229)	*p*
Sex			
Female	29 (21.2)	108 (78.8)	0.147
Male	48 (28.4)	121 (71.6)	
Age (years)			
< 30	2 (50.0)	2 (50.0)	0.614
30-50	13 (25.0)	39 (75.0)	
51-70	47 (26.0)	134 (74.0)	
71-90	15 (21.7)	54 (78.3)	
Exercise habits			
No	4 (12.9)	27 (87.1)	0.097
Yes	73 (26.5)	202 (73.5)	
Dietary structure			
Balanced diet	53 (29.4)	127 (70.6)	0.039*
Dietary bias	24 (19.0)	102 (81.0)	
BMI			
Lean	6 (30.0)	14 (70.0)	0.925
Normal	39 (24.4)	121 (75.6)	
Overweight	27 (24.4)	82 (75.2)	
Corpulent	5 (29.4)	12 (70.6)	
Marital status			
No spouse	6 (19.4)	25 (80.6)	0.432
Have a spouse	71 (25.8)	204 (74.2)	
Education level			
Elementary	23 (20.4)	90 (79.6)	0.292
Secondary	27 (26.5)	75 (73.5)	
University	27 (29.7)	64 (70.3)	
Working status			
Retire	47 (22.2)	165 (77.8)	0.087
Incumbency	22 (36.1)	39 (63.9)	
Other	8 (24.2)	25 (75.8)	
Cancer type			
Gastrointestinal cancer	23 (24.7)	70 (75.3)	0.886
Lung cancer	20 (24.7)	61 (75.3)	
Breast cancer	11 (22.0)	39 (78.0)	
Other	23 (28.0)	59 (72.0)	
Tumor status			
Tumor-free survival	32 (27.8)	83 (72.2)	0.405
Survival with tumor	45 (23.6)	146 (76.4)	
Current cancer stage			
I	7 (9.1)	17 (7.4)	0.989
II	11 (14.3)	32 (14)	
III	17 (22.1)	54 (23.6)	
IV	40 (51.9)	119 (52.0)	
Unknown	2 (2.6)	7 (3.1)	
Stage of treatment			
Just diagnosed	3 (21.4)	11 (78.6)	0.837
Peri-western medical treatment period	61 (24.8)	185 (75.2)	
Follow-up period	13 (28.3)	33 (71.7)	
Current therapy			
No	6 (42.9)	8 (57.1)	0.237
Chemotherapy	33 (29.7)	78 (70.3)	
Radiotherapy	10 (22.7)	34 (77.3)	
Targeted therapy	6 (24.0)	19 (76.0)	
TCM treatment	22 (19.6)	90 (80.4)	

*p- value< 0.05, p- value was based on Chi-square.

As shown in **Table 3**, in terms of both anxiety (*p* = 0.003) and depression (*p*< 0.001) states and anxiety (*p*< 0.001) and depression (*p*< 0.001) scores, the difference comparison results showed a statistically significant association between anxiety, depression, and CRF incidence.

**Table 3:** Comparison of differences between non-CRF and CRF in HADS.

		Non-CRF (*n* =77)	CRF (*n* = 229)	*p*
HADS-states *n* (%)	Anxiety (*n*=38)	2 (5.3)	36 (94.7)	0.003*
	Depression (*n*=79)	2 (2.5)	77 (97.5)	< 0.001*
HADS-score Mean (SD)	Anxiety	1.22 (2.14)	3.75 (3.88)	< 0.001*
	Depression	2.44 (2.48)	6.72 (4.95)	< 0.001*

HADS-states: HADS-A, HADS-D ≥ 8 points; *p– value< 0.05, p– value was based.

As shown in **Table 4**, there was a significant correlation between aldosterone levels and CRF occurrence (*p*< 0.001), however, no significant correlation was found between ACTH, COR, Renin and CRF (*p* > 0.05).

**Table 4:** Comparison of differences between in Serum hormone levels.

	Non-CRF (*n* = 17)	CRF (*n* = 55)	*p*
ACTH Mean (SD)	31.5 (15.08)	36.67 (21.46)	0.548
Renin Mean (SD)	11.8 (9.36)	14.21 (10.62)	0.406
COR Mean (SD)	14.2 (4.06)	16.07 (22.41)	0.720
ALD Mean (SD)	136.84 (40.2)	169.96 (65.12)	0.032*

*p– value< 0.05, p– value was based on Mann-Whitney; SD, standard deviation.

### 3.3 Comparison of different levels of fatigue

As **Table 5** shows, there was no statistically significant difference in demographic, sociological characteristics between groups with different levels of fatigue (*p* > 0.05), however, KPS were significantly associated with fatigue level (*p*< 0.001).

**Table 5:** Comparison of different levels of fatigue in general information.

	Mild fatigue (*n* = 94)	Moderate fatigue (*n* = 121)	Severe fatigue (*n* = 14)	*p*
Sex				
Female	39 (36.1)	61 (56.5)	8 (7.4)	0.320
Male	55 (45.5)	60 (49.6)	6 (5.0)	
Age (years)				
< 30	1 (50.0)	1 (50.0)	0 (0)	0.602
30-50	17 (43.6)	19 (48.7)	3 (7.7)	
51-70	54 (40.3)	75 (56.0)	5 (3.7)	
71-90	22 (40.7)	26 (48.1)	6 (11.1)	
Exercise habits				
No	8 (29.6)	17 (62.0)	2 (7.4)	0.440
Yes	86 (42.6)	104 (51.5)	12 (5.9)	
Dietary structure				
Balanced diet	49 (38.6)	69 (54.3)	9 (7.1)	0.613
Dietary bias	45 (44.1)	52 (51.0)	5 (4.9)	
BMI				
Lean	5 (35.7)	9 (64.3)	0 (0)	0.968
Normal	52 (43.0)	60 (49.6)	9 (7.4)	
Overweight	32 (39.0)	46 (56.1)	4 (4.9)	
Corpulent	5 (41.7)	6 (50.0)	1 (8.3)	
Marital status				
No spouse	7 (28.0)	15 (60.0)	3 (12.0)	0.222
Have a spouse	87 (42.6)	106 (52.0)	11 (5.4)	
Education level				
Elementary	39 (43.3)	47 (52.2)	4 (4.4)	0.845
Secondary	30 (40.0)	37 (49.3)	8 (10.7)	
University	25 (39.1)	37 (57.8)	2 (3.1)	
Working status				
Retire	66 (40.0)	89 (53.9)	10 (6.1)	0.921
Incumbency	19 (48.7)	18 (46.2)	2 (5.1)	
Other	19 (48.7)	18 (46.2)	2 (5.1)	
Cancer type				
Gastrointestinal cancer	31 (44.3)	32 (45.7)	7 (10.0)	0.181
Lung cancer	26 (42.6)	32 (52.5)	3 (4.9)	
Breast cancer	13 (33.3)	24 (61.5)	2 (5.1)	
Other	24 (40.7)	33 (55.9)	2 (3.4)	
Tumor status				
Tumor-free survival	39 (47.0)	40 (48.2)	4 (4.8)	0.368
Survival with tumor	55 (37.7)	81 (55.5)	10 (6.8)	
Current cancer stage				
I	9 (9.6)	7 (5.8)	1 (7.1)	0.400
II	12 (12.8)	19 (15.7)	1 (7.1)	
III	25 (26.6)	27 (22.3)	2 (14.3)	
IV	44 (46.8)	66 (54.5)	9 (64.3)	
Unknown	4 (4.3)	2 (1.7)	1 (7.1)	
Stage of treatment				
Just diagnosed	3 (27.3)	8 (72.7)	0 (0)	0.080
Peri-western medical treatment period	72 (38.9)	99 (53.5)	14 (7.6)	
Follow-up period	19 (57.6)	14 (42.4)	0 (0)	
Current therapy				
No	2 (25.0)	4 (50.0)	2 (25.0)	0.068
Chemotherapy	34 (43.6)	42 (53.8)	2 (2.6)	
Radiotherapy	9 (26.5)	25 (73.5)	0 (0)	
Targeted therapy	8 (42.1)	10 (52.6)	1 (5.3)	
TCM treatment	41 (45.6)	40 (44.4)	9 (10.0)	

p- value was based on Kruskal-Wallis.

Similar to the results of the between-group difference comparison for CRF or not, anxiety and depression (anxiety and depression states and anxiety and depression scores) were significantly correlated with fatigue level (*p*< 0.001) (**Table 6**).

**Table 6:** Comparison of different levels of fatigue in HADS.

		Mild fatigue (*n* = 94)	Moderate fatigue (*n* = 121)	Severe fatigue (*n* = 14)	*p*
HADS-states *n* (%)	Anxiety (*n*=38)	2 (2.1)	26 (21.5)	8 (57.1)	< 0.001*
	Depression (*n*=79)	14 (14.8)	51(42.1)	12 (85.7)	< 0.001*
HADS-score Mean (SD)	Anxiety	2 (2.20)	4.3 (3.53)	10.79 (6.04)	< 0.001*
	Depression	4.22 (3.19)	7.57 (4.43)	16.21 (5.21)	< 0.001*

HADS-states: HADS-A, HADS-D ≥ 8 points; *p- value< 0.05, p- value was based on Kruskal-Wallis.

In terms of serum hormone levels, there was no significant correlation that was found between ACTH, Renin, COR, ALD and different levels of fatigue (*p* > 0.05) (**Table 7**).

**Table 7:** Comparison of different levels of fatigue in serum hormone levels.

	Mild fatigue (*n* = 27)	Moderate fatigue (*n* = 26)	Severe fatigue (*n* = 2)	*p*
ACTH Mean (SD)	39.18 (18.51)	35.52 (24.39)	23.97 (20.76)	0.239
Renin Mean (SD)	15.75 (11.39)	12.66 (10.19)	13.64 (0)	0.419
COR Mean (SD)	18.79 (31.45)	13.95 (5.82)	7 (8.82)	0.471
ALD Mean (SD)	176.8 (69.48)	167.24 (61.63)	112.98 (23.82)	0.194

p- value was based on Kruskal-Wallis, SD: standard deviation.

### 3.4 Multivariable analysis

In addition to including variables with *p<* 0.05 in the difference comparison into multivariable logistic regression analysis, control variables such as age, gender, BMI, stage of disease treatment, and previous and current treatment modalities were also included. Because of the strong correlation between KPS scores and fatigue, they were excluded from the regression. As **model 1** shows, BMI ≥ 28 kg/m^2^ was negatively associated with the occurrence of CRF (OR = 0.011, 95% CI 0.001-0.770, *p* = 0.037), and higher depression scores (OR = 1.811, 95% CI 1.301-2.720, *p* = 0.001) or the higher the level of aldosterone the higher the risk of CRF (OR =1.025, 95% CI 1.005-1.045, *p* = 0.015). As **model 2** shows, relative to male, female had an increased risk of fatigue (OR = 11.069, 95% CI 2.147-57.083, *p* = 0.004). The model also showed that the higher the anxiety, depression score, and aldosterone levels, the higher the risk of increased fatigue (OR = 1.496, 95% CI 1.128-1.985, *p* = 0.005; OR = 1.379, 95% CI 1.144-1.661, *p* = 0.001; OR = 1.012, 95%CI 1.002-1.022, *p* = 0.024) (**Table 8**).

**Table 8:** Multivariable analysis using logistic regressions.

Model 1: Binary regression model of CRF occurrence							
Factors	B	Standard error (SE)	Wald χ2	*p*	OR	OR 95% CI	
BMI							
Lean	·	·	6.690	0.082	·	·	·
Normal	-2.636	1.973	1.785	0.182	0.072	0.001	3.424
Overweight	0.136	1.188	0.013	0.909	1.145	0.112	11.755
Corpulent	-4.510	2.168	4.328	0.037	0.011	0.001	0.770
Depression	0.632	0.188	11.296	0.001	1.811	1.301	2.720
ALD	0.024	0.010	5.963	0.015	1.025	1.005	1.045
Constant	-5.365	1.870	8.227	0.004	0.005		
Model 2: Ordinal logistic regression of increased fatigue							
Factors	B	Standard error (SE)	Wald χ2	*p*	OR	OR 95% CI	
Female	2.404	0.837	8.252	0.004	11.069	2.147	57.083
Anxiety	0.403	0.144	7.799	0.005	1.496	1.128	1.985
Depression	0.321	0.095	11.391	0.001	1.379	1.144	1.661
ALD	0.012	0.005	5.090	0.024	1.012	1.002	1.022

Model 1: Binary logistic regression is performed based on “fatigue or not” as the dependent variable.

Model 2: Ordinal logistic regression is performed based on “different levels of fatigue” as the dependent variable.

"·" represents Lean as the reference category compared to Normal, Overweight, and Corpulent.

p- value< 0.05.

## 4 Discussion

Our study revealed that CRF is a prevalent issue among various types of cancer survivors, yet it has not received sufficient attention in China (30). We included 17 kinds of tumors, among which gastrointestinal tumors, lung cancer, and breast cancer are the most common cancers with a high incidence of CRF. No significant differences were observed across cancer types, aligning with Wang XS’s findings (31). Besides, our study showed that the prevalence of fatigue in 75% of cancer survivors indicates that nearly two-thirds of these individuals will require fatigue management. But the reality is that only 10.5% of patients have heard of CRF, and less than 5% seeking active interventions. These findings emphasize the critical need to integrate CRF education and awareness programs into clinical settings, ensuring that patients are adequately informed about this debilitating condition.

Similar to previously studies (32, 33), our study found that gender, emotion state, and BMI were influential factors for CRF. Specifically, women, as well as individuals with higher depression scores, were found to be at a higher risk of CRF and increased fatigue, which aligns with existing studies (29, 34).Women tend to be more susceptible to anxiety and depression when facing changes in their body function and quality of life after cancer treatment (35). Hence, the NCCN panel recommended that in addition to regular fatigue assessment, emotional assessment be integrated into patient care at all stages—from admission to hospitalization and post-discharge—to prevent or alleviate fatigue in vulnerable populations (36). Due to pharmacologic interventions had limited study data in CRF patients and depression or anxiety, often presents as a cluster of symptoms alongside fatigue. As a result, interventions that affect multiple systems, such as complementary therapies, may be more recommended (37). Indeed, psychosocial, exercise, and mind-body interventions appear to be more beneficial for CRF than pharmacotherapy (38), perhaps because these approaches have effects on a range of biobehavioral processes relevant for fatigue.

Our study introduces some novel insights into the relationship between dietary habits, BMI, and CRF. Patients with dietary biases, such as those following a vegan or meat-based diet and those consuming a high-salt diet, appeared to be more prone to CRF. Furthermore, our multivariate regression analysis suggests that a BMI of ≥28 kg/m² may reduce the risk of CRF. These findings raise intriguing questions about the potential links between nutrition, obesity, and CRF. While previous studies have generally associated obesity with poorer cancer survival rates and increased fatigue, recent research has proposed the concept of an “*obesity paradox*” in cancer patients (39). Based on this point of view, many studies have proved that early obesity is related to a higher survival rate of cancer patients. For example, a meta-analysis showed that colorectal cancer patients with higher BMI had lower mortality than normal-weight patients (40), and another cross-sectional study showed that CRF development, inflammatory markers and fatty acid levels were mainly associated with class II (35.0-39.9 kg/m^2^) and class III (≥40.0kg/m^2^) obesity (41). In addition, a study that intervened breast cancer patients with a Mediterranean diet and exercise for 1 year showed that a traditional Mediterranean diet and weight loss reduced the level of fatigue in the patients (42). Thus, the NCCN guidelines state that cancer and treatment can interfere with dietary intake, nutrition consultation may be helpful in managing the nutritional deficiencies that result from anorexia, diarrhea, nausea, and vomiting (43). Our study contributes to this discussion, highlighting the complex relationship between body weight, diet, and CRF. Large RCTs are needed to determine the impact of nutrition therapy on CRF in the future.

Chronic stressors like anxiety and depression can affect CRF by influencing the hypothalamic-pituitary-adrenal (HPA) axis (19, 44). Through a negative feedback control loop, the HPA axis normally regulates the release of stress-related hormones (i.e., cortisol and ACTH) in response to physical or psychological stress (45). Our study suggests that high aldosterone levels are an important risk factor for CRF and increased fatigue. According to earlier research, patients with depression have dysregulation of the RAAS system, and their plasma aldosterone levels are 2.77 times higher than those of healthy people without psychological problems (46). Recent studies have pointed out that activation of the RAAS system is closely related to tumor proliferation and angiogenesis, and tumor progression is usually the direct cause of fatigue (47). Considering the key roles of the RAAS system and aldosterone in tumor progression and depression, we speculate that aldosterone may be an important predictor of the differential experience of fatigue. Because it was only a cross-sectional survey, the link between these findings and the CRF is exploratory. However, our findings and previous research indicate that CRF is linked to emotional distress (such as anxiety and depression) and stress-related hormones. Identifying the emotional distress and neuroendocrine alterations underlying CRF is an important focus for future research and has significant implications for interventions.

Our study has several limitations, in order to know the overall incidence of CRF in different tumors, so we did not limit the tumor species included, which may make the results lack relevance in some aspects. However, our analysis revealed that CRF is a common symptom among patients with different tumors, which is in line with our original idea to draw attention to CRF by investigating its prevalence. CRF is a multidimensional and subjective symptom, so we used the scale as an assessment tool to better reflect the patient’s immediate fatigue or psychological condition. On the other hand, because of the small sample size and single-center cross-sectional design, all the factors we assessed were obtained at one time point, and the relationship between CRF and these factors was exploratory, and the potential association needs to be confirmed by further prospective studies. In addition, because the scale we used was an examiner-rating scale, we could not exclude that patient modified some of the results; although the scale was a Chinese version, semantic and cultural differences were still found in the specific application, and two of the patients declined to complete the final survey because the scale entries were difficult to comprehend during the survey.

## 5 Conclusions

In conclusion, our study showed that gender, BMI, emotional distress, and aldosterone may be influential factors in the differential experience of fatigue. This underscores the importance of comprehensive assessments that consider fatigue, emotional health, and nutritional status, which are essential for preventing and reducing CRF and improving quality of life in the growing population of cancer survivors. Furthermore, the identification of neural processes and neuroendocrine alterations that influence fatigue may help in the development of targeted interventions for those most in need.

## Data availability statement

The raw data supporting the conclusions of this article will be made available by the authors, without undue reservation.

## Ethics statement

The studies involving humans were approved by The Medical Ethics Committee of Xiyuan Hospital, China Academy of Chinese Medical Sciences (Approval number: 2021XLA027; Approval date: March 26, 2021). The studies were conducted in accordance with the local legislation and institutional requirements. The participants provided their written informed consent to participate in this study.

## Author contributions

YX, SG contributed to the study conception and design. Support in sample size estimation and choice of suitable measuring instruments/questionnaires was provided by DY and KL, SG, LG, JW, XG, LF, JS contributed to the data collection. SG performed the data analysis and wrote the paper YX, XZ, A.L revised the paper. All authors read and approved the final manuscript.
